# Semantic Function in Mild Cognitive Impairment

**DOI:** 10.3389/fpsyg.2019.03041

**Published:** 2020-01-22

**Authors:** Vanessa Taler, Laura Monetta, Christine Sheppard, Avery Ohman

**Affiliations:** ^1^School of Psychology, University of Ottawa, Ottawa, ON, Canada; ^2^Bruyère Research Institute, Ottawa, ON, Canada; ^3^Département de Réadaptation, Université Laval, Québec, QC, Canada; ^4^Centre de Recherche de l’Institut Universitaire en Santé Mentale de Québec, Québec, QC, Canada; ^5^Interdisciplinary School of Health Sciences, University of Ottawa, Ottawa, ON, Canada

**Keywords:** picture naming, lexical access, semantic features, neuropsychological assessment, mild cognitive impairment

## Abstract

It is well-established that semantic deficits are observed in mild cognitive impairment (MCI). However, the extent of impairment in different aspects of semantic function remains unclear, and may be influenced by the tasks used to assess performance. In the present study, people with MCI and cognitively healthy older adults completed a series of tasks assessing lexical access, retrieval, and recognition of semantic information, using different input and output modalities. Control participants outperformed people with MCI in almost all tasks, with the greatest deficits observed in picture naming tasks. This finding is interpreted as reflecting greater deficits in lexical access and/or access to the phonological and orthographic lexicon, and less severe deficits in retrieval and recognition of semantic feature and associative knowledge. In a subset of tasks, relatively greater impairment was also observed in biological compared to man-made items. From a clinical perspective, these results suggest that, while it is preferable that a full semantic battery be included in neuropsychological assessment, in cases where shorter testing time is necessary, picture naming is the task most likely to reveal deficits in people with MCI.

## Introduction

Mild cognitive impairment (MCI), first defined in Petersen et al. (1999), refers to an impairment in both objective and subjective cognitive function in the absence of frank dementia. MCI is a major risk factor for development of dementia, with most studies showing a risk of development of dementia of 20–40% (10–15% per annum) (for a review, see Roberts and Knopman, 2013). While MCI was originally defined in terms of impairment in memory function, criteria were subsequently revised to include subtypes of MCI that present with or without memory impairment (amnestic and non-amnestic MCI), as well as deficits in just one or in multiple cognitive domains (single- vs. multiple-domain MCI) (Petersen et al., 2001).

One area where deficits are observed in MCI is in semantic function. Semantic knowledge refers to our knowledge about objects, facts, and concepts, and includes our knowledge of words and their meanings. It is a critical component for most cognitive functions (Patterson and Hodges, 1995) and according to the semantic hub model (Patterson et al., 2007; Lambon Ralph and Patterson, 2008), it is represented in an amodal fashion. This model of semantic representation is based on empirical data showing that the amodal representations forming the “semantic hub” are anatomically supported by anterior regions of the temporal lobes bilaterally. Moreover, this model implies that a true semantic deficit should be present irrespective of the modality tested (e.g., oral, written, visual). Thus, a multi-modal interactive evaluation of semantic function will allow better assessment of semantic capacity.

Several studies have demonstrated that significant declines in semantic memory are consistent and common in MCI and early stages of Alzheimer’s disease (AD) (Ahmed et al., 2008; Barbeau et al., 2012; Salmon, 2012). Semantic memory is stable in healthy older adults (OAs) but shows a similar pattern of decline in people with amnestic MCI and AD (Adlam et al., 2006; Joubert et al., 2010; Taler et al., 2016). This pattern of decline is possibly due to degradation of semantic representations, as well as selective deficits in the selection, manipulation, and retrieval of semantic information (Joubert et al., 2010; Taler et al., 2016). However, while the semantic deficit in MCI is well-established (for a review, see Taler and Phillips, 2008), the exact nature of the impairment remains unclear (Adlam et al., 2006), and performance is affected by the tasks used for assessment (Brandt and Manning, 2009).

A distinction has also been observed in AD between performance on semantic tasks where the items tested are biological (e.g., animals, fruits) and those where the items tested are artifacts (i.e., non-biological; e.g., tools and clothing). Category-specific deficits have been reported in AD and MCI, with most studies finding greater impairment for biological than artifact items. Whatmough et al. (2003) examined semantic function in a group of participants with MCI and varying severities of AD, and reported a category effect that was present in all participants whose naming scores were not within the range of cognitively healthy OAs. The effect was found to be greater with worsening anomia. Taler et al. (2016) similarly report greater impairment on biological items than artifacts in people with MCI on a semantic feature task.

It has been suggested that this category-specific deficit arises as a result of the distribution of semantic features across items. Whatmough et al. (2003) point out that biological items possess a higher number of intercorrelated features than artifacts. That is, animals are more likely to closely resemble other animals compared to, for example, tools; thus, performance on semantic tasks requiring unique identification will be higher for artifacts due to their greater distinctiveness. Brambati et al. (2006) used voxel-based morphometry to identify the neural bases of naming performance in 152 patients with different neurodegenerative diseases. They found no category-specific effect when familiarity was controlled for. However, naming performance for biological and artifact items was correlated with different brain regions: for biological items, naming performance was correlated with gray matter volume in the medial portion of the right anterior temporal pole, while for artifacts, naming performance was correlated with volume in the left posterior middle temporal gyrus. They argue that the defining features of biological and artifact items differ, with the former being defined by shared sensory features, while the latter is defined by action-related features, and that their anatomical segregation arises from the different neural subsystems used to process each type of feature.

When conducting cognitive assessments in MCI, guidelines recommend that language be among the cognitive domains evaluated (Albert et al., 2011), and indeed the most commonly used tasks to assess semantic function have been verbal. These include picture naming tasks such as the Boston Naming Test (Kaplan et al., 1983), as well as verbal fluency tasks in which participants must name as many items as possible within 60 s that conform to a given criterion (e.g., animals, words that start with the letter F). However, semantic function encompasses both verbal and non-verbal abilities, and exclusively verbal tasks are therefore not ideal for semantic assessment. In order to gain a true picture of a person’s semantic function, following the semantic hub model (Patterson et al., 2007), performance must be assessed using tasks that tap multiple input and output modalities (e.g., words, pictures, speaking, writing, pointing). In this way, it is possible to rule out effects that are related to the input and output channels, and identify deficits that are truly semantic in nature, which should be present across all testing modalities. The use of different tasks also allows us to distinguish between semantic and executive deficits: many tasks typically used to assess semantic function (e.g., category fluency) also place heavy demands on executive functions and may therefore not be ideal as stand-alone tasks to identify semantic deficits.

In the present study, we had groups of people with MCI and cognitively healthy OAs complete a series of tasks assessing semantic function using a variety of input and output modalities, and examining both biological and artifact items. Given previous research indicating that semantic function is impaired in MCI, we predicted that deficits should be observed in all tasks, with relatively greater deficits in biological compared to artifact items, as observed in previous research. Given that each task taps different aspects of semantic function, we also aimed to identify the tasks where semantic impairments are most apparent, shedding light on the nature of the semantic impairment in MCI as well as providing evidence to guide clinical assessment of semantic function in this population.

## Materials and Methods

### Participants

Two groups of participants took part in this study: cognitively healthy OAs (*n* = 41) and people with a diagnosis of MCI (*n* = 38). Participants with MCI were recruited from the Bruyère Memory Program in Ottawa, Canada, and had received a formal diagnosis of MCI from a geriatrician or neurologist on the basis of the Petersen criteria (Petersen et al., 1999). Because MCI subtype was not consistently included as part of the diagnosis, this information is not used in the current study. MCI participants underwent neuroimaging and blood work to rule out alternative diagnoses, and were living independently in the community. Cognitively healthy participants were recruited from the Ottawa community via word of mouth and through print advertisements. All participants spoke English as the primary language and did not have any neurologic or psychiatric history, other than that under study. All participants completed a neuropsychological battery examining cognitive status, executive function, semantic function, verbal learning, and working memory. This battery included the Montreal Cognitive Assessment (MoCA, Nasreddine et al., 2005), the Pyramids and Palm Trees Test (Howard and Patterson, 1992), the California Verbal Learning Test – II (Delis et al., 2000), the Boston Naming Test (Kaplan et al., 1983), the Wisconsin Card Sorting Test (WCST, Grant and Berg, 1948), the digit span, letter-number sequencing, and digit symbol subtasks of the Wechsler Adult Intelligence Scale – III (Wechsler, 1997), the Stroop task (Stroop, 1935), and animal and letter (FAS) fluency tasks. Demographic and neuropsychological information is provided in Table 1. This study received ethical approval from the Research Ethics Boards at the Bruyère Research Institute and the University of Ottawa in Ottawa, Canada.

**TABLE 1:** Participant demographic information and performance on neuropsychological tasks.

	**OA (*n* = 41; 13 males)**	**MCI (*n* = 38; 21 males)**
Age	73.32 ± 3.72	75.76 ± 7.16
Education	15.51 ± 2.67	15.21 ± 3.11
MoCA (/30)∗∗∗	27.29 ± 1.69	21.43 ± 3.34
PPT word (/52)	50.90 ± 1.07	50.15 ± 1.71
PPT picture (/52)∗∗	50.97 ± 1.04	50.09 ± 1.69
Digit symbol oral (/110)∗∗∗	51.51 ± 9.31	41.29 ± 10.81
Digit symbol written (/110)∗∗∗	42.63 ± 7.28	35.24 ± 9.58
CVLT∗∗∗	58.90 ± 7.70	33.40 ± 8.53
Animal fluency	14.83 ± 4.81	14.34 ± 3.65
Fluency (FAS)∗∗	43.74 ± 10.59	35.40 ± 11.75
WCST (/6)∗∗∗	3.71 ± 1.33	2.24 ± 1.07
Stroop (words)∗∗	98.02 ± 16.88	84.91 ± 16.11
Stroop (colors)∗∗∗	64.98 ± 10.14	53.72 ± 10.53
Stroop (interference)∗∗∗	35.52 ± 8.33	26.24 ± 9.35
BNT (/60)	47.56 ± 6.69	47.00 ± 6.80
Digit span forward (/15)	10.14 ± 2.07	10.06 ± 2.09
Digit span backward (/15)	6.43 ± 2.27	6.43 ± 2.28
Letter number sequencing (/21)∗∗∗	10.44 ± 2.40	7.57 ± 2.44

*∗∗*p* < 0.01; ∗∗∗*p* < 0.001. BNT, Boston Naming Test; CVLT, California Verbal Learning Test; MCI, mild cognitive impairment; MoCA, Montreal Cognitive Assessment; OA, cognitively healthy older adult; PPT, Pyramids and Palm Trees; WCST, Wisconsin Card Sorting Test.*

### Materials

#### Task 1: Picture Naming

Participants completed picture naming tasks in two different modalities: spoken picture naming (Task 1a) and written picture naming (Task 1b). In each subtask, participants were presented with 18 visual images on a computer screen and asked to either orally name the image (Task 1a) or write the name of the image on a response sheet (Task 1b). Stimuli for each subtask comprised six action items, six artifact items, and six biological items; stimuli did not overlap between Tasks 1a and 1b. Stimuli were matched for frequency across category and task using norms (HAL frequency) from the English Lexicon Project (Balota et al., 2007). Biological and artifact items were matched for length, and action items were slightly orthographically shorter overall. (Stimuli for this and all other tasks are provided in the Supplementary Material.)

#### Task 2: Associative Matching

Participants completed two subtasks assessing matching of associated images, one with pictures as the input modality (Task 2a) and one with written words (Task 2b). Each subtask comprised 12 trials, six biological and six artifact. In each trial, participants saw either an image (Task 2a) or a word (Task 2b) at the top of a computer screen with four items below, one of which was associatively related to the top image or word (i.e., they share a contextual link, e.g., shark-ocean). They were asked to identify the associated image or word by pointing. Stimuli did not overlap between the two subtasks, and were matched for length, and for frequency across category and task using norms (HAL frequency) from the English Lexicon Project.

#### Task 3: Common Feature Identification

Participants completed two subtasks assessing the capacity to identify semantic features that two items have in common. In Task 3a (Generation), participants heard two words, and were asked to provide the most specific common feature (e.g., tiger-zebra, where the correct response is that both have stripes). If the participant failed to provide a response or provided a response that was too general (e.g., “both are animals”), they were prompted once (“is there another way that these two things are similar?”). Task 3b (Multiple Choice) also entailed identifying the way in which two items were similar, but in this case the participant was asked to select from among four options. Input was both written and spoken: the participant was provided with a written sheet which was read aloud by the experimenter. Each subtask contained 12 trials: six pairs of biological items and six pairs of artifact items. Stimuli did not overlap between the two subtasks, and were matched for length and frequency across category and task using norms (HAL frequency) from the English Lexicon Project. (For further details on design and administration of Tasks 3a and 3b, see Taler et al., 2016.)

#### Task 4: Semantic Feature Questions

Participants were asked a series of questions with “yes” or “no” answers (e.g., are monkeys hairy?). A total of 24 questions were asked, 12 pertaining to biological items and 12 to artifact items. For each category, half of the questions were related to encyclopedic/functional characteristics of the item, and half to perceptual characteristics.

#### Analyses

Age, education, and performance on each test in the neuropsychological battery were analyzed using *t*-tests. Sex was compared using a chi-square test. To analyze performance on the tasks in the semantic battery, we first conducted a series of ANOVAs to examine overall group performance in each of the tasks completed. Because errors in picture naming can reflect deficits at multiple levels, in order to better understand the nature of the observed deficit, we conducted error analyses for Tasks 1a and 1b (spoken and written picture naming). Each error was categorized as visual (e.g., “cup” for “thimble”), phonological (e.g., “dragon” for “dragonfly”), circumlocution (e.g., “man is playing with the balls circularly” for “juggling”), no answer, or semantic. Semantic errors were further categorized as associative (e.g., “music” for “record player”), category coordinate (e.g., “goat” for “sheep”), or category superordinate (e.g., “bird” for “peacock”).

## Results

The groups were matched for age, sex, and education. Significant differences were observed in most of the tasks in the neuropsychological battery (Table 1). Overall group performance on the semantic battery is shown in Table 2. Data were first analyzed using a series of ANOVAs. For Tasks 1a and 1b, we conducted 2 (group) × 3 (biological vs. artifact vs. action) ANOVAs. For the remaining tasks (2a, 2b, 3a, 3b, and 4), we conducted 2 (group) × 2 (biological vs. artifact) ANOVAs. Interactions were decomposed using LSD *post hoc* tests.

**TABLE 2:** Overall task performance.

**Task**	**Subtask**	**Stimulus type**	**OA**	**MCI**
Task 1: Picture naming	Spoken	Action	99.59 ± 2.60	95.42 ± 9.98
		Biological	93.09 ± 14.90	66.25 ± 24.89
		Artifact	89.42 ± 9.69	80.42 ± 19.57
	Written	Action	90.00 ± 11.82	86.25 ± 14.06
		Biological	98.33 ± 5.06	91.25 ± 14.12
		Artifact	91.25 ± 11.31	75.83 ± 18.85
Task 2: Associative matching	Picture–picture	Biological	99.19 ± 3.63	95.42 ± 8.43
		Artifact	94.71 ± 7.85	91.25 ± 9.23
	Word–word	Biological	99.59 ± 2.60	98.33 ± 5.06
		Artifact	100.00 ± 0.00	97.08 ± 7.44
Task 3: Common feature identification	Generation	Biological	76.86 ± 24.73	68.80 ± 28.14
		Artifact	92.87 ± 14.00	87.60 ± 13.09
	Multiple choice	Biological	93.90 ± 11.03	82.50 ± 19.23
		Artifact	94.71 ± 8.69	95.42 ± 9.98
Task 4: Semantic questions		Biological	94.71 ± 8.69	90.38 ± 10.21
		Artifact	95.93 ± 6.21	93.80 ± 8.27

*Accuracy is given in percentage (mean ± standard deviation). OA, cognitively healthy older adult; MCI, mild cognitive impairment.*

### Task 1: Picture Naming

#### Spoken Picture Naming

Overall, cognitively healthy OAs performed better than people with MCI [main effect of group, *F*(1,79) = 29.50, *p* < 0.001, ${\text{η}}_{\text{p}}^{2}$ = 0.27]. Action items elicited the highest performance, followed by artifact and biological items, which did not differ [main effect of category, *F*(2,158) = 39.93, *p* < 0.001, ${\text{η}}_{\text{p}}^{2}$ = 0.34]. A significant interaction was observed between group and stimulus type [*F*(2,158) = 16.93, *p* < 0.001, ${\text{η}}_{\text{p}}^{2}$ = 0.18]. OAs scored significantly better on action than artifact (*p* ≤ 0.001) and biological items (*p* = 0.044), which did not differ (*p* = 0.25). People with MCI performed best on action items, followed by artifact and then biological items (*p* ≤ 0.001 for all comparisons).

#### Written Picture Naming

Overall, OAs performed better than people with MCI [main effect of group, *F*(1,78) = 19.61, *p* < 0.001, ${\text{η}}_{\text{p}}^{2}$ = 0.20]. Biological items elicited the highest performance, followed by action and artifact items, which did not differ [main effect of category, *F*(2,156) = 17.74, *p* < 0.001, ${\text{η}}_{\text{p}}^{2}$ = 0.19]. An interaction was observed between group and category [*F*(2,186) = 5.01, *p* = 0.008, ${\text{η}}_{\text{p}}^{2}$ = 0.06], whereby OAs performed better on biological than action (*p* = 0.001) and artifact items (*p* = 0.01), which did not differ (*p* = 0.66). People with MCI performed better on biological than action items (*p* = 0.05), and better on action than artifact items (*p* < 0.001).

### Task 2: Associative Matching

#### Picture–Picture Matching

Older adults outperformed people with MCI [main effect of group, *F*(1,79) = 6.57, *p* = 0.01, ${\text{η}}_{\text{p}}^{2}$ = 0.08], and performance was higher on biological than artifact items [main effect of category, *F*(1,79) = 22.00, *p* < 0.001, ${\text{η}}_{\text{p}}^{2}$ = 0.22]; however, no significant interaction between category and group was observed [*F*(1,79) = 0.03].

#### Word–Word Matching

Older adults outperformed people with MCI [main effect of group, *F*(1,79) = 5.93, *p* = 0.02, ${\text{η}}_{\text{p}}^{2}$ = 0.07]; however, there was no effect of category [*F*(1,79) = 0.53] nor any interaction between category and group [*F*(1,79) = 2.04].

### Task 3: Common Feature Identification

#### Generation

The group difference in performance on this task did not reach significance [*F*(1,79) = 3.61, *p* = 0.06]. Overall, performance was better on artifact than biological items [main effect of category, *F*(1,79) = 32.02, *p* < 0.001, ${\text{η}}_{\text{p}}^{2}$ = 0.29]. No interaction was observed between group and category [*F*(1,79) = 1.06].

#### Multiple Choice

Older adults outperformed people with MCI [main effect of group, *F*(1,79) = 5.91, *p* = 0.02, ${\text{η}}_{\text{p}}^{2}$ = 0.07]. A main effect of category was also observed [*F*(1,79) = 14.12, *p* < 0.001, ${\text{η}}_{\text{p}}^{2}$ = 0.15]; performance was higher overall on artifact compared to biological items. An interaction between group and category was also observed [*F*(1,79) = 10.97, *p* = 0.001, ${\text{η}}_{\text{p}}^{2}$ = 0.12]. Performance was higher on artifact than biological items in people with MCI (*p* < 0.001) but not healthy OAs (*p* = 0.75).

### Task 4: Semantic Feature Questions

Overall, OAs outperformed people with MCI on this task [main effect of group, *F*(1,78) = 4.55, *p* = 0.04, ${\text{η}}_{\text{p}}^{2}$ = 0.06]. A main effect of category was observed [*F*(1,78) = 4.22, *p* = 0.04, ${\text{η}}_{\text{p}}^{2}$ = 0.05]; performance was higher overall on artifact than biological items. There was no interaction between group and category [*F*(1,78) = 0.95].

#### Summary

Results by task are summarized in Table 3. Overall, healthy OAs outperformed people with MCI in all tasks except Task 3a (generation of common semantic features). The largest effect sizes were observed in the picture naming tasks. Greater impairment in biological compared to artifact stimuli was observed in two tasks, Task 1a (spoken picture naming) and Task 3b (selection of common semantic feature).

**TABLE 3:** Summary of performance by task in cognitively healthy older adults and people with MCI.

**Task**	**Subtask**	**Input modality**	**Output modality**	**Group effect?**	**Differential group effect by category?**
Task 1: Picture naming	Spoken	Picture	Spoken	Yes, ${\text{η}}_{\text{p}}^{2}$ = 0.27	OA: act > art = bio MCI: act > art > bio
	Written	Picture	Written	Yes, ${\text{η}}_{\text{p}}^{2}$ = 0.20	OA: bio > act = art MCI: bio > act > art
Task 2: Associative matching	Picture–picture	Picture	Pointing	Yes, ${\text{η}}_{\text{p}}^{2}$ = 0.08	No
	Word–word	Written	Pointing	Yes, ${\text{η}}_{\text{p}}^{2}$ = 0.07	No
Task 3: Common feature identification	Generation	Spoken	Spoken	No	No
	Multiple choice	Written and spoken	Spoken	Yes, ${\text{η}}_{\text{p}}^{2}$ = 0.07	OA: art = bio MCI: art > bio
Task 4: Semantic feature questions		Spoken	Spoken	Yes, ${\text{η}}_{\text{p}}^{2}$ = 0.06	No

*act, action; art, artifact; bio, biological; OA, older adult; MCI, mild cognitive impairment.*

### Error Analyses

Overall error type by group, test type, and stimulus type, are shown in Tables 4A (spoken naming), B (written naming). For the purposes of the present analysis, any error type that represented <5% of the total errors was excluded, resulting in the retention of three error types in the analysis: no answer, semantic–category coordinate, and semantic–associative.

**TABLE 4:** **(A)** Error types by group, spoken naming task and **(B)** error types by group, written naming task.

**Group**	**Item type**	**No answer**	**Semantic—category coordinate**	**Semantic—associative**
**(A)**				

Older adults	Action	0	25	1
	Artifact	0	12	9
	Biological	0	4	0
MCI	Action	0	30	1
	Artifact	6	22	28
	Biological	4	16	0

**(B)**				

Older adults	Action	1	0	0
	Artifact	2	24	0
	Biological	4	8	0
MCI	Action	0	2	1
	Artifact	2	35	2
	Biological	26	37	0

Because the total number of errors was low, we combined the spoken and written naming data for the purposes of statistical analyses. We conducted a series of 2 × 2 chi-squares within each error type to compare performance of OAs and people with MCI on action, artifact, and biological items. A significant effect was found only for semantic–category coordinate errors in artifact vs. biological items (χ^2^ = 7.42, *p* = 0.006). That is, people with MCI were more likely than OAs to commit semantic–category coordinate errors in biological items (e.g., “flamingo” for peacock).

## Discussion

Overall, the results indicate semantic deficits in the MCI sample included here compared to the cognitively healthy OAs. OAs outperformed people with MCI in six of the seven tasks included here, as well as in standardized semantic tasks (Boston Naming Test and Pyramids and Palms – pictures, but not Pyramids and Palms – words). These results are consistent with a large literature indicating semantic deficits in MCI (e.g., Adlam et al., 2006; Ahmed et al., 2008; Balthazar et al., 2008; Joubert et al., 2010; Barbeau et al., 2012; Taler et al., 2016; Gallant et al., 2019). Semantic deficits were observed in tasks where the input modality was either verbal (written or spoken) or non-verbal (pictures), and in tasks where the output modality was verbal (spoken or written) or non-verbal (pointing), suggesting a true semantic deficit rather than an impairment in one input or output modality. This result is in accord with the semantic hub model (Patterson et al., 2007), which holds that a true semantic deficit should be observable irrespective of the modality used to evaluate it. The only task in which differences were not observed between people with MCI and healthy OAs was the common semantic feature generation task (e.g., how is a tiger like a zebra). We note that performance for healthy OAs was quite low in this task, particularly for biological items (76% correct), suggesting that task difficulty likely plays a role in the absence of a group difference.

Effect sizes differed across tasks, with the largest effect sizes in picture naming, and small to null effects in the remaining tasks. Of the tasks used here, picture naming (both spoken and written) places the greatest demands on lexical access and phonological/orthographic lexicons, while the remaining tasks require recognition of associative information (Task 2), retrieval (Tasks 3a and 4), or recognition (Task 3b) of semantic feature information. The present results thus shed light on the nature of the semantic impairment in MCI. Importantly, the greatest deficits were not observed in the most difficult tasks (as assessed by performance of cognitively healthy OAs on the task). Rather, it appears that lexical access is affected in MCI to a greater extent than retrieval or recognition of semantic and associative information. This is in line with previous work indicating anomia in MCI (Dudas et al., 2005; Adlam et al., 2006; Balthazar et al., 2008, 2010). Recent work from our group examining naming errors in MCI suggests that the deficit appears to originate from impairments both in lexical access and in the semantic system (Gallant et al., 2019).

A second possible account for the greater naming deficits observed in MCI relates to the role of specific brain regions in naming performance. Previous research has suggested that naming is sustained in MCI and AD by the left anterior temporal lobe (Frings et al., 2011), the region where the amodal semantic hub is located in Patterson et al.’s (2007) model. The finding that people with MCI show greater deficits in naming tasks, compared to the other tasks included in the semantic battery, is therefore consistent with the postulation that a true (amodal) semantic deficit occurs in MCI. Future research should investigate the neural bases of performance in other semantic tasks in people with MCI in order to determine the extent to which left anterior temporal dysfunction drives the deficits observed here.

It is possible that the relative preservation of performance on semantic feature tasks, compared to a lexical access task, reflects a different timecourse of impairment in MCI, particularly given evidence that tasks tapping semantic feature and associative information are impaired in people with AD. However, the current cross-sectional study cannot definitively establish the timecourse of impairment. Future longitudinal research should be undertaken to resolve this question. We also note that we did not have access to information about premorbid intelligence levels or cognitive reserve status, a limitation of the present study.

With respect to category-specific deficits, consistent with our hypotheses, we found differential group effects for biological items in two of the seven tasks: spoken picture naming and multiple choice common feature identification. In both spoken picture naming and multiple choice common feature identification, people with MCI, but not cognitively healthy OAs, exhibited lower performance on biological than artifact items. This finding is consistent with previous research indicating category-specific impairment in biological items in MCI (e.g., Whatmough et al., 2003; Taler et al., 2016). We also found that people with MCI were more likely than cognitively healthy OAs to produce semantic–category coordinate errors on biological than artifact items in a picture naming task, suggesting that the category-specific deficit is truly semantic in nature rather than related to visual, phonological, or other impairments. It is notable that Brambati et al. (2006) found correlations between right anterior temporal lobe volume and naming performance on biological items but not artifacts. Thus, the present findings suggest that a greater impairment in naming than other semantic tasks in MCI, in combination with a category-specific deficit for biological items, could arise from bilateral anterior temporal dysfunction.

In sum, the results of the present study indicate semantic deficits in MCI that are stronger in tasks that also tap lexical access and the phonological or orthographic lexicon (spoken and written picture naming), and weaker in tasks requiring retrieval or recognition of semantic feature information. The findings also provide some evidence for a stronger deficit in biological than manmade items. From a clinical perspective, these results suggest that picture naming is the task most likely to reveal deficits in MCI; thus, under time constraints, spoken/written picture naming is the most appropriate task for assessment of semantic function in this population.

## Data Availability Statement

The datasets generated for this study are available on request to the corresponding author.

## Ethics Statement

The studies involving human participants were reviewed and approved by the Bruyère Research Ethics Board and the University of Ottawa Research Ethics Board. The patients/participants provided their written informed consent to participate in this study.

## Author Contributions

VT: conceptualization, stimulus development, data analysis, and manuscript preparation. LM: conceptualization, stimulus development, and manuscript contribution. CS: stimulus development, data analysis, and manuscript contribution. AO: data collection and analysis and manuscript contribution.

## Conflict of Interest

The authors declare that the research was conducted in the absence of any commercial or financial relationships that could be construed as a potential conflict of interest.

## References

[B1] Adlam A. L. R. Bozeat S. Arnold R. Watson P. Hodges J. R. (2006). Semantic knowledge in mild cognitive impairment and mild Alzheimer’s disease. *Cortex* 42 675–684. 10.1016/S0010-9452(08)70404-0 16909626

[B2] Ahmed S. Arnold R. Thompson S. A. Graham K. S. Hodges J. R. (2008). Naming of objects, faces and buildings in mild cognitive impairment. *Cortex* 44 746–752. 10.1016/j.cortex.2007.02.002 18472044

[B3] Albert M. S. DeKosky S. T. Dickson D. Dubois B. Feldman H. H. Fox N. C. (2011). The diagnosis of mild cognitive impairment due to Alzheimer’s disease: recommendations from the National Institute on Aging-Alzheimer’s association workgroups on diagnostic guidelines for Alzheimer’s disease. *Alzheimer’s Dement.* 7 270–279. 10.1016/j.jalz.2011.03.008 21514249 PMC3312027

[B4] Balota D. A. Yap M. J. Cortese M. J. Hutchison K. A. Kessler B. Loftis B. (2007). The english lexicon project. *Behav. Res. Methods* 39 445–459. 10.3758/bf03193014 17958156

[B5] Balthazar M. L. F. Cendes F. Damasceno B. P. (2008). Semantic error patterns on the Boston Naming Test in normal aging, amnestic mild cognitive impairment, and mild Alzheimer’s disease: is there semantic disruption? *Neuropsychology* 22 703–709. 10.1037/a0012919 18999343

[B6] Balthazar M. L. F. Yasuda C. L. Cendes F. Damasceno B. P. (2010). Learning, retrieval, and recognition are compromised in aMCI and mild AD: are distinct episodic memory processes mediated by the same anatomical structures? *J. Int. Neuropsychol. Soc.* 16 205–209. 10.1017/S1355617709990956 19835661

[B7] Barbeau E. J. Didic M. Joubert S. Guedj E. Koric L. Felician O. (2012). Extent and neural basis of semantic memory impairment in mild cognitive impairment. *J. Alzheimer’s Dis.* 28 823–837. 10.3233/JAD-2011-110989 22112550

[B8] Brambati S. M. Myers D. Wilson A. Rankin K. P. Allison S. C. Rosen H. J. (2006). The anatomy of category-specific object naming in neurodegenerative diseases. *J. Cogn. Neurosci.* 18 1644–1653. 10.1162/jocn.2006.18.10.1644 17014369

[B9] Brandt J. Manning K. J. (2009). Patterns of word-list generation in mild cognitive impairment and Alzheimer’s disease. *Clin. Neuropsychol.* 23 870–879. 10.1080/13854040802585063 19301196 PMC2857742

[B10] Delis D. Kramer J. Kaplan E. Ober B. (2000). *California Verbal Learning Test*, 2nd Edn San Antonio, TX: Psychological Corporation.

[B11] Dudas R. B. Clague F. Thompson S. A. Graham K. S. Hodges J. R. (2005). Episodic and semantic memory in mild cognitive impairment. *Neuropsychologia* 43 1266–1276. 10.1016/j.neuropsychologia.2004.12.005 15949511

[B12] Frings L. Kloppel S. Teipel S. Peters O. Frolich L. Pantel J. (2011). Left anterior temporal lobe sustains naming in Alzheimer’s dementia and mild cognitive impairment. *Curr. Alzheimer Res.* 8 893–901. 10.2174/156720511798192673 21592050

[B13] Gallant M. Lavoie M. Hudon C. Monetta L. (2019). Analyse des erreurs de dénomination dans le vieillissement normal, le trouble cognitive léger et la maladie d’Alzheimer. *Revue Canadienne d’orthophonie et d’audiologie* 43 95–108.

[B14] Grant D. A. Berg E. (1948). A behavioral analysis of degree of reinforcement and ease of shifting to new responses in a Weigl-type card-sorting problem. *J. Exp. Psychol.* 38 404–411. 10.1037/h0059831 18874598

[B15] Howard D. Patterson K. (1992). “*Pyramids and Palm Trees: a Test of Semantic Access.* Bury St Edmunds: Thames Valley Publishing Company. 10.1037/h0059831

[B16] Joubert S. Brambati S. M. Ansado J. Barbeau E. J. Felician O. Didic M. (2010). The cognitive and neural expression of semantic memory impairment in mild cognitive impairment and early Alzheimer’s disease. *Neuropsychologia* 48 978–988. 10.1016/j.neuropsychologia.2009.11.019 19954747

[B17] Kaplan E. Goodglass H. Weintraub S. (1983). *Boston Naming Test.* Philadelphia, PA: Lea & Febiger.

[B18] Lambon Ralph M. A. Patterson K. (2008). Generalization and differentiation in semantic memory. *Ann. N. Y. Acad. Sci.* 1124 61–76. 10.1196/annals.1440.006 18400924

[B19] Nasreddine Z. S. Phillips N. A. Bedirian V. Charbonneau S. Whitehead V. Collin I. (2005). The Montreal Cognitive Assessment, MoCA: a brief screening tool for mild cognitive impairment. *J. Am. Geriat. Soc.* 53 695–699. 10.1111/j.1532-5415.2005.53221.x 15817019

[B20] Patterson K. Hodges J. (1995). “Disorders of semantic memory,” in *Handbook of Memory Disorders*, eds Baddeley A. D. Wilson B. A. Watts F. N. (Chichester: John Wiley.), 167–186.

[B21] Patterson K. Nestor P. J. Rogers T. T. (2007). Where do you know what you know? The representation of semantic knowledge in the human brain. *Nat. Rev. Neurosci.* 8 976–987. 10.1038/nrn2277 18026167

[B22] Petersen R. C. Doody R. Kurz A. Mohs R. C. Morris J. C. Rabins P. V. (2001). Current concepts in mild cognitive impairment. *Arch. Neurol.* 58 1985–1992. 10.1001/archneur.58.12.1985 11735772

[B23] Petersen R. C. Smith G. E. Waring S. C. Ivnik R. J. Tangalos E. G. Kokmen E. (1999). Mild cognitive impairment: clinical characterization and outcome. *Arch. Neurol.* 56 303–308. 10.1001/archneur.56.3.303 10190820

[B24] Roberts R. Knopman D. S. (2013). Classification and epidemiology of MCI. *Clin. Geriat. Med.* 29 753–772. 10.1016/j.cger.2013.07.003 24094295 PMC3821397

[B25] Salmon D. P. (2012). Loss of semantic knowledge in mild cognitive impairment. *Am. J. Psychiatry* 169 1226–1229. 10.1176/appi.ajp.2012.12101262 23212051

[B26] Stroop J. (1935). Studies of interference in serial verbal reactions. *J. Exp. Psychol.* 18 643–662. 10.1037/h0054651

[B27] Taler V. Phillips N. (2008). Language performance in Alzheimer’s disease and mild cognitive impairment: a comparative review. *J. Clin. Exp. Neuropsychol.* 30 501–556. 10.1080/13803390701550128 18569251

[B28] Taler V. Voronchikhina A. Gorfine G. Lukasik M. (2016). Knowledge of semantic features in mild cognitive impairment. *J. Neurolinguistics* 38 56–70. 10.1016/j.jneuroling.2015.11.002

[B29] Wechsler D. (1997). *Wechsler Adult Intelligence Scale*, 3rd Edn San Antonio, TX: The Psychological Corporation.

[B30] Whatmough C. Chertkow H. Murtha S. Templeman D. Babins L. Kelner N. (2003). The semantic category effect increases with worsening anomia in Alzheimer’s type dementia. *Brain Lang.* 84 134–147. 10.1016/S0093-934X(02)00524-2 12537956

