# Supplementary material for: Semantic Function in Mild Cognitive Impairment
Source: Front Psychol. 2020 Jan 22;10:3041. doi: 10.3389/fpsyg.2019.03041 (PMC6987430; doi:10.3389/fpsyg.2019.03041)
Supplement: Supplementary file 1 [file Table_1.DOCX]

**Appendix**

a) b)


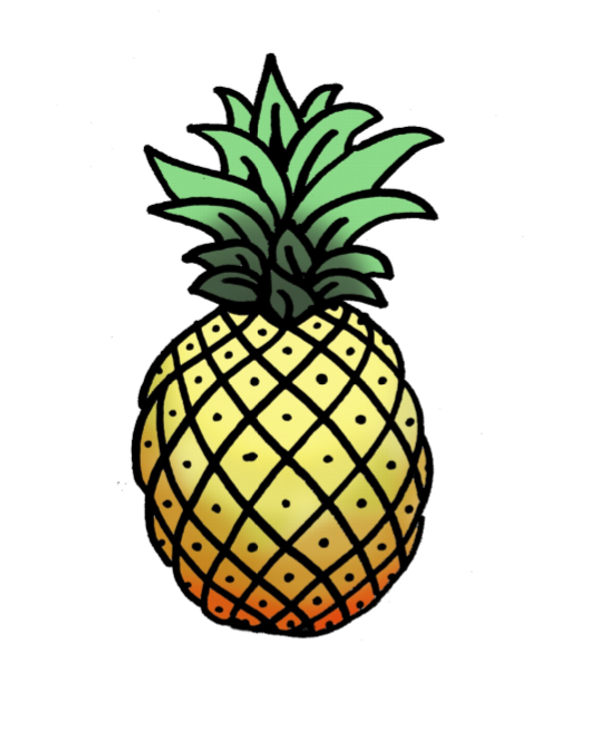

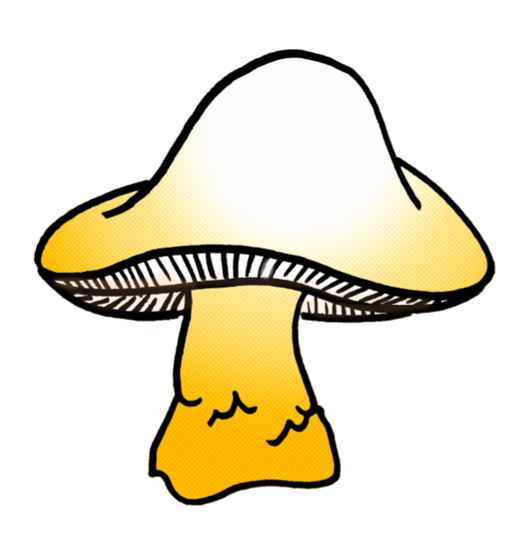


*Figure 1*. Examples from Task 1 – Picture Naming. Task 1a requires an oral response (pineapple) and Task 1b requires a written response (mushroom).

a) b)


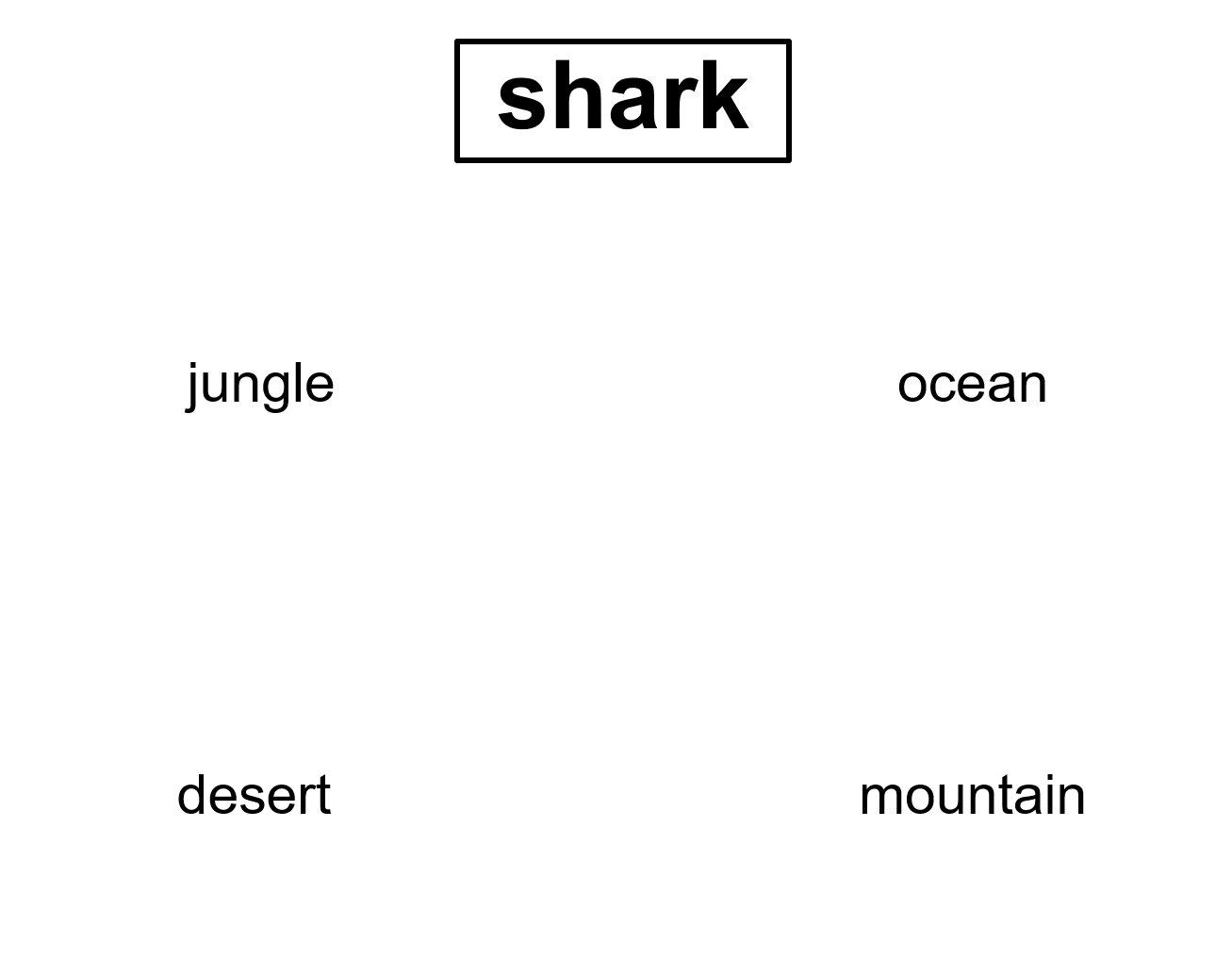

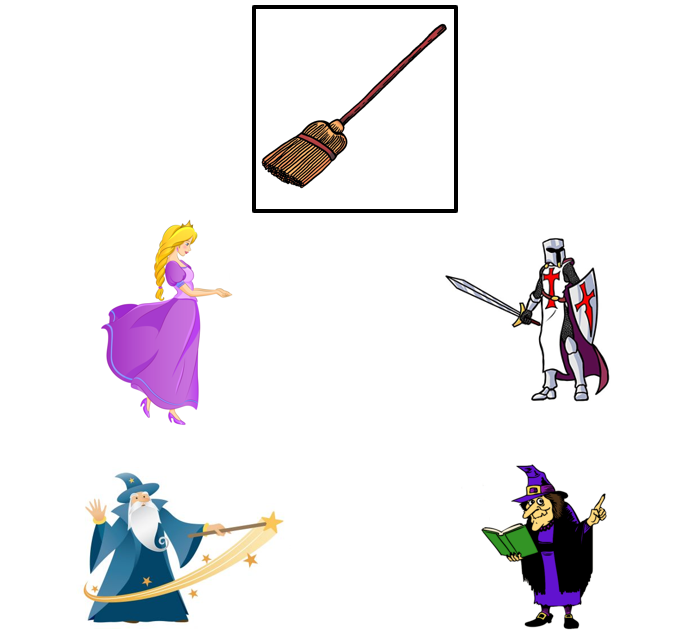


*Figure 2*. Example from Task 2 – Associative Matching. Task 2a is word-word association (shark-ocean) and Task 2b is picture-picture association (broom-witch)

TABLE 1. Tasks of Semantic Function with Stimulus Items in the Neuropsychological Battery.

| Task | Subtask | Stimulus type | Stimulus |
| --- | --- | --- | --- |
| Task 1: Picture naming | Spoken | Action | Crawl |
|  |  |  | Knit |
|  |  |  | Swim |
|  |  |  | Shave |
|  |  |  | Yawn |
|  |  |  | Juggle |
|  |  | Biological | Pepper |
|  |  |  | Peacock |
|  |  |  | Seahorse |
|  |  |  | Pineapple |
|  |  |  | Dragonfly |
|  |  |  | Duck |
|  |  | Artifact | Salt Shaker |
|  |  |  | Axe |
|  |  |  | Well |
|  |  |  | Doorknob |
|  |  |  | Watering Can |
|  |  |  | Sled |
|  | Written | Action | Drink |
|  |  |  | Smile |
|  |  |  | Skate |
|  |  |  | Stretch |
|  |  |  | Wave |
|  |  |  | Cook |
|  |  | Biological | Mushroom |
|  |  |  | Sheep |
|  |  |  | Lobster |
|  |  |  | Butterfly |
|  |  |  | Pumpkin |
|  |  |  | Squirrel |
|  |  | Artifact | Thimble |
|  |  |  | Record Player |
|  |  |  | Spinning Wheel |
|  |  |  | Ring |
|  |  |  | Spool |
|  |  |  | Bow |
| Task 2: Associative matching | Picture-picture | Biological | Horse |
|  |  |  | Chicken |
|  |  |  | Milk |
|  |  |  | Banana |
|  |  |  | Caterpillar |
|  |  |  | Elephant |
|  |  | Artifact | Broom |
|  |  |  | Nail |
|  |  |  | Crown |
|  |  |  | Pillow |
|  |  |  | Net |
|  |  |  | Helmet |
|  | Word-word | Biological | Shark |
|  |  |  | Pine |
|  |  |  | Apple |
|  |  |  | Rabbit |
|  |  |  | Deer |
|  |  |  | Bee |
|  |  | Artifact | Sleigh |
|  |  |  | Tweezers |
|  |  |  | Vase |
|  |  |  | Ring |
|  |  |  | Ambulance |
|  |  |  | Glasses |
| Task 3: Common feature identification | Generation | Biological | Eggplant-Plum |
|  |  |  | Chihuahua-Hummingbird |
|  |  |  | Owl-Raccoon |
|  |  |  | Crow-Panther |
|  |  |  | Greyhound-Cheetah |
|  |  |  | Pig-Flamingo |
|  |  | Artifact | Banjo-Guitar |
|  |  |  | Lasso-Net |
|  |  |  | Telescope-Glasses |
|  |  |  | Anchor-Dumbbell |
|  |  |  | Piano-Accordion |
|  |  |  | Racquet-Bat |
|  | Multiple choice | Biological | Bee-Wasp |
|  |  |  | Calf-Puppy |
|  |  |  | Mink-Rabbit |
|  |  |  | Penguin-Panda |
|  |  |  | Leopard-Dalmatian |
|  |  |  | Carrot-Pumpkin |
|  |  | Artifact | Envelope-Napkin |
|  |  |  | Bagpipe-Kilt |
|  |  |  | Paperclip-Stapler |
|  |  |  | Broom-Toothbrush |
|  |  |  | Telephone-Alarm |
|  |  |  | Helicopter-Airplane |
| Task 4: Semantic questions |  | Biological | Are monkeys hairy? |
|  |  |  | Do parakeets have stripes? |
|  |  |  | Do sheep have beards? |
|  |  |  | Can whales jump out of the water? |
|  |  |  | Do snails have antennae? |
|  |  |  | Do kangaroos come from China? |
|  |  |  | Do tangerines need to be peeled before eating them? |
|  |  |  | Is lettuce usually cooked? |
|  |  |  | Are squirrels nocturnal? |
|  |  |  | Do hippos have trunks? |
|  |  |  | Are cranberries eaten with turkey? |
|  |  |  | Does garlic have skin? |
|  |  | Artifact | Does a cup usually have a lid? |
|  |  |  | Do surfboards float? |
|  |  |  | Does a clarinet have a mouthpiece? |
|  |  |  | Do toasters produce steam? |
|  |  |  | Are canoes used with paddles? |
|  |  |  | Do pianos have strings? |
|  |  |  | Is a spatula round? |
|  |  |  | Are earmuffs usually used in summer? |
|  |  |  | Are limousines usually long? |
|  |  |  | Is a soccer ball usually thrown? |
|  |  |  | Is a buggy usually pulled by horses? |
|  |  |  | Does a wheelbarrow usually have two wheels? |
